# Preparation of Hollow Niobium Oxide Nanospheres with Enhanced Catalytic Activity for Oxidative Desulfurization

**DOI:** 10.3390/nano12071106

**Published:** 2022-03-28

**Authors:** Yong Wang, Lei Ren, Zifeng Li, Feng Xin

**Affiliations:** 1 Sinopec Research Institute of Petroleum Processing, Beijing 100083, China; renlei.ripp@sinopec.com (L.R.); lizf.ripp@sinopec.com (Z.L.); 2 School of Chemical Engineering and Technology, Tianjin University, Tianjin 300350, China; xinf@tju.edu.cn

**Keywords:** three-dimensional mesoporous carbon, hollow niobium oxide nanosphere, dibenzothiophene, oxidative desulfurization

## Abstract

Hollow niobium oxide nanospheres were successfully synthesized by using prepared three-dimensional (3D) mesoporous carbon as the hard template. The 3D mesoporous carbon materials were prepared by using histidine as the carbon source and silica microspheres as the hard template. The samples were characterized by XRD, BET, SEM, TEM and other methods. The results show that the prepared niobium oxide nanospheres have a hollow spherical structure with an outer diameter of about 45 nm and possess a high specific surface area of 134.3 m^2^·g^−1^. Furthermore, the 3D mesoporous carbon materials have a typical porous structure with a high specific surface area of 893 m^2^·g^−1^. The hollow niobium oxide nanospheres exhibit high catalytic activity in oxidative desulfurization. Under optimal reaction conditions, the DBT conversion rate of the simulated oil is as high as 98.5%. Finally, a possible reaction mechanism is proposed.

## 1. Introduction

In the past few decades, increasing attention has been paid to environmental pollution problems due to rapid industrial development, the increase in the number of vehicles and the combustion of fossil fuels. Among all environmental problems, the emission of harmful SO_x_ derivatives from the combustion of sulfur-containing organic compounds in fuel oils poses a great threat to the environment [1]. Today, in many countries, the sulfur content in fuel oils is strictly limited to less than 10 ppm, and increasingly stringent fuel quality standards worldwide have led to a great deal of research on efficient desulfurization technologies to reduce the sulfur content in fuel oils [2,3].

Hydrodesulfurization has long been used as the main desulfurization method in the petroleum refining industry, especially for the removal of aliphatic and non-cyclic sulfur-containing compounds [4]. However, hydrodesulfurization is less efficient for the removal of some aromatic sulfur compounds, such as dibenzothiophene and its derivatives [5]. To obtain ultra-clean lighter oils, higher temperatures and pressures are inevitably required, leading to higher operating costs [6]. Therefore, there is an increasing interest in non-hydrodesulfurization methods, including extraction desulfurization [7], adsorption desulfurization [8], oxidation desulfurization [9] and biodesulfurization [10]. Among them, oxidative desulfurization is considered one of the most promising and economical methods due to its mild operating conditions, low cost and greenness.

In recent years, hollow nanostructures have attracted great interest due to their specific structure and properties [11]. There are two commonly used methods for the synthesis of hollow structures: the soft template method and hard template method [12,13,14,15]. The soft template method is a one-step synthesis method that generally uses amphiphilic surfactants directly as soft templates to self-assemble mesoporous structured materials. So far, only amorphous or semi-crystalline hollow metal oxides have been synthesized by the soft template method [16]. The hard template method entails introducing the precursor of the material to a hard template with a special pore structure, and the reaction occurs in the pores. Hollow materials can be obtained by using the domain-limiting effect of the hard template, which has the advantages of easy removal and structural stability.

In this study, hollow niobium oxide nanospheres were prepared by the hard template method. Three-dimensional (3D) mesoporous carbon materials were prepared by using histidine as the carbon source and prepared silica microspheres as the hard template. The effect of different mass ratios of silica and histidine on the morphology of the synthesized 3D mesoporous carbon materials was analyzed. Subsequently, the hollow niobium oxide nanospheres were prepared by a vacuum-assisted method using the prepared 3D mesoporous carbon as the hard template and niobium pentachloride as the precursor, and the prepared samples were applied in the catalytic oxidation desulfurization reaction of the simulated oil.

## 2. Materials and Methods

### 2.1. Reagents and Chemicals

Tetraethyl orthosilicate (TEOS), lysine, histidine, absolute ethanol, n-octane, potassium hydroxide, niobium pentachloride, benzothiophene (BT), dibenzothiophene (DBT), 4, 6-dimethyldibenzothiophene (4, 6-DMDBT), N-methylpyrrolidone (NMP), hydrogen peroxide (30 wt%), etc., were purchased from Sinopharm Chemical Reagent Co., Ltd. (Shanghai, China). All chemicals were of analytical grade and used without any further purification.

### 2.2. Preparation of Catalyst

#### 2.2.1. Preparation of 3D Mesoporous Carbon

**Figure 1:**
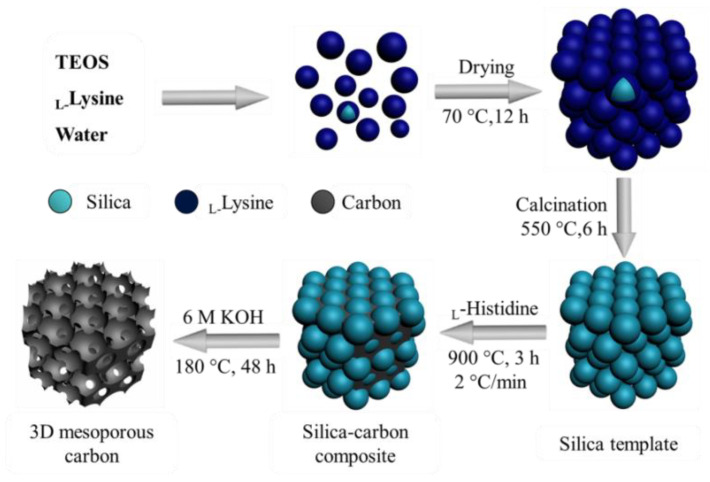
The preparation process of 3D mesoporous carbon materials.

Three-dimensional mesoporous carbon materials were prepared by using histidine as the carbon source and prepared silica microspheres as the hard template. The typical preparation process of 3D mesoporous carbon materials is shown in Figure 1.

1. First, 70 mg of lysine was dissolved in 69.3 g of deionized water. Subsequently, 5.4 g of tetraethyl orthosilicate was added to the lysine solution and then transferred to a water bath and stirred at 60 °C for 24 h. The stirring speed was maintained at 500 rpm.
2. A total of 10 g of the above sol was added to 40 g of water. Subsequently, 10.4 g of tetraethyl orthosilicate was added to the lysine solution and then transferred to a water bath and stirred at 60 °C for 48 h. The stirring speed was maintained at 500 rpm.
3. The obtained silica nanosphere sols were poured into a Petri dish and dried in an oven at 70 °C for 12 h to allow the spheres to settle and remove water. The obtained solids were calcined in air at 550 °C for 6 h to remove the organic components and to obtain silica templates.
4. Histidine (0.75~2.0 g) and silica nanospheres (1 g) were mixed evenly by grinding. The mixture was subsequently transferred to a tube furnace and directly heated to 900 °C for 3 h under a nitrogen atmosphere.
5. The calcined solid was ground into powder and dispersed in potassium hydroxide solution (70 mL, 6 mol/L). Then, the mixed solution was transferred to a 100 mL Teflon-lined autoclave and heated to 180 °C for 48 h. The mixed solution was naturally cooled to room temperature and filtered, washed with deionized water until the filtrate was nearly neutral and then dried at 60 °C for 12 h. Finally, 3D mesoporous carbon was obtained, denoted as 3DmC-900.

#### 2.2.2. Preparation of Hollow Niobium Oxide Nanospheres

**Figure 2:**
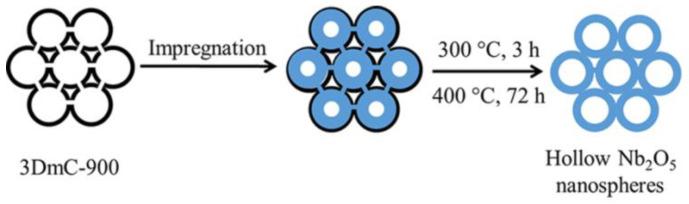
The preparation process of hollow niobium oxide nanospheres.

The preparation process of hollow niobium oxide nanospheres is shown in Figure 2.

1. First, 0.1 g of 3DmC-900 was added to an excess of niobium pentachloride ethanol solution (0.75 g/mL), stirred until a uniform mixture was obtained and then allowed to stand for 12 h at room temperature.
2. The mixed solution was filtered under vacuum to remove excess precursors, and then the obtained solid powder was dried in a vacuum drying oven for 3 h.
3. The dried solid powder was calcined at 300 °C under a nitrogen atmosphere for 3 h to convert niobium pentachloride into niobium oxide and then calcined at 400 °C under an air atmosphere for 72 h to remove the carbon template. Finally, the hollow niobium oxide nanospheres were obtained.

### 2.3. Characterization of Materials

X-ray diffraction (XRD) measurements were recorded in the range of 10° to 80° (2θ) on a Rigaku Ultima IV diffractometer using Cu-Kα radiation (Tokyo, Japan). Scanning electron microscopy (SEM) images were obtained using a Hitachi S-4800 electron microscope (Tokyo, Japan). The elemental composition of the samples was measured by an energy-dispersive X-ray spectrometer (EDX) attached to a scanning electron microscope (Tokyo, Japan). The high-resolution transmission electron microscopy (HRTEM) images of the samples were taken on a JEOL JSM-3010 microscope (Tokyo, Japan). N_2_ adsorption/desorption isotherms were examined on a Quantachrome Quadrasorb SI apparatus at 77 K (Boynton Beach, FL, USA).

### 2.4. Catalytic Oxidation Desulfurization Experiments

A certain amount of sulfur compounds (BT, DBT and 4, 6-DMDBT) was dissolved in n-octane to prepare simulated oil with a corresponding S-content of 500 mg/kg. The hollow niobium oxide nanosphere catalyst, simulated oil and NMP were added to a three-neck flask, and the reaction mixture was heated to a constant temperature. Then, the reaction mixture was stirred vigorously for 15 min for extraction. H_2_O_2_ was added to carry out the oxidative desulfurization reaction at a stirring rate of 400 r/min. The desulfurization activity of the catalyst was expressed by the removal rate of DBT.

## 3. Results and Discussion

### 3.1. Morphology and Structure of the Catalysts

#### 3.1.1. 3D Mesoporous Carbon

**Figure 3:**
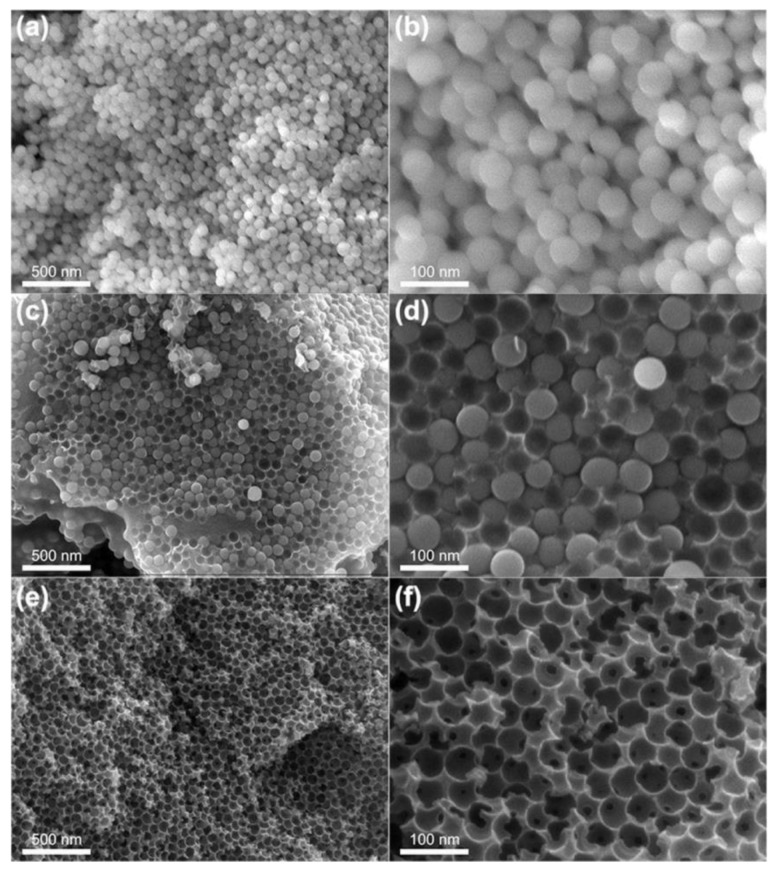
(**a**,**b**) SEM images of silica nanospheres, (**c**,**d**) SEM images of silica/carbon composite, and (**e**,**f**) SEM images of 3D mesoporous carbons templated from silica nanospheres.

Figure 3a,b show the SEM images of the calcined silica nanospheres. The prepared silica nanospheres are uniform in size with a smooth surface and perfect spherical shape, and the spheres are closely packed. After calcination, the size of the spheres was reduced to a diameter of about 50 nm. As seen in Figure 3e, the resulting 3D mesoporous carbon completely replicates the silica template after the removal of the template. It can be seen from Figure 3f that in the 3D mesoporous carbon, each spherical pore is also interconnected with adjacent pores in three dimensions through small pores, which is attributed to the contact points between the closely packed silica spheres. Figure S1 displays the SEM images of carbon materials prepared with different mass ratios of silica to histidine. The suitable mass ratio of silica to histidine is 1:1.5. Therefore, we chose the 3D mesoporous carbon prepared with this ratio as the template for preparing niobium oxide nanospheres.

**Figure 4:**
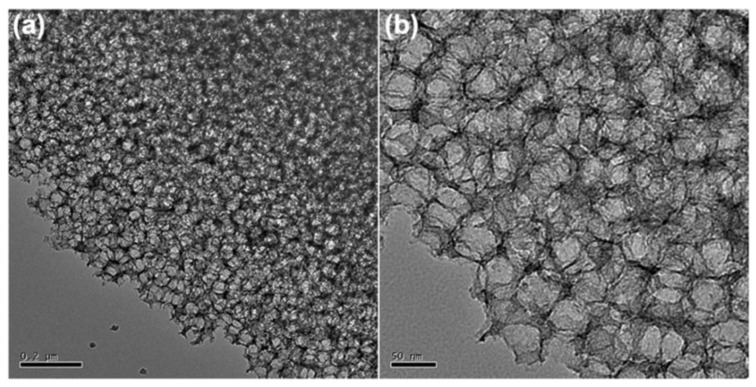
TEM images of 3D mesoporous carbon templated from silica nanospheres (**a**,**b**).

The TEM images of the three-dimensional mesoporous carbon prepared with silica nanospheres as a template are shown in Figure 4a,b. The size of the mesopores in the 3D mesoporous carbon is about 45 nm. These sizes are somewhat smaller than those of the silica spheres used as templates. This may be due to the reduction in the size of the silica spheres during the high-temperature carbonization process, which caused the radial shrinkage of the carbon source [17]. The analysis results of TEM further indicate that the inverted structure of the closely packed silica template was successfully transformed into 3D mesoporous carbon.

**Figure 5:**
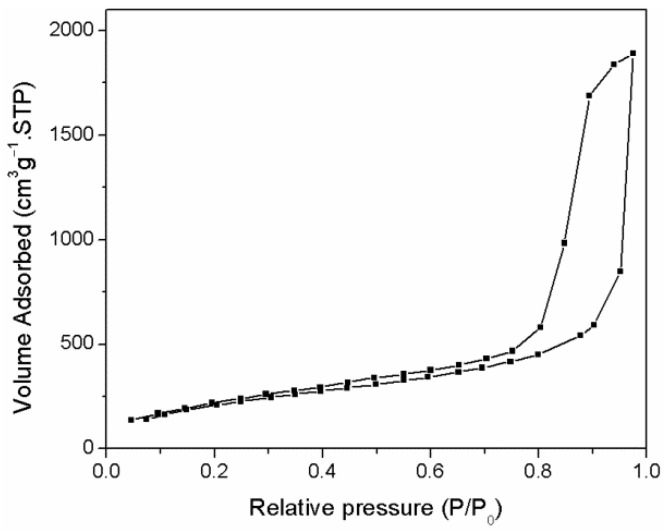
N_2_ adsorption/desorption isotherms of the 3D mesoporous carbon.

The texture properties of 3D mesoporous carbon were studied using the nitrogen/adsorption measurement method (Figure 5). The prepared 3D mesoporous carbon sample exhibits type I hysteresis at low pressure, representing the microporosity of the amorphous carbon matrix. It belongs to the type IV isotherm at high relative pressure and forms an obvious H1 hysteresis loop with nearly vertical and almost parallel adsorption and desorption branches, indicating that the 3D mesoporous carbon has an abundant mesoporous structure [18]. The specific surface area of the 3D mesoporous carbon calculated from the multipoint BET model is as high as 893 m^2^·g^−1^.

#### 3.1.2. Hollow Nb_2_O_5_ Nanospheres

**Figure 6:**
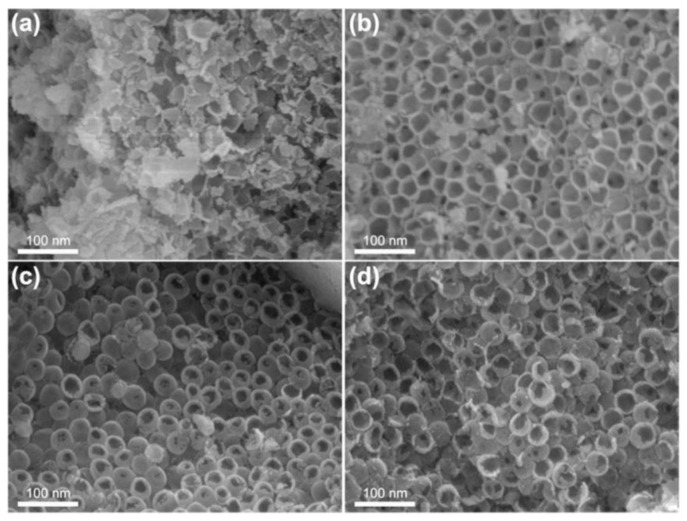
SEM images of hollow Nb_2_O_5_ nanospheres obtained by different concentrations of NbCl_5_ ethanol solution: (**a**) 0.25 g/mL, (**b**) 0.5 g/mL, and (**c**) 0.75 g/mL; (**d**) SEM images of hollow mesoporous Nb_2_O_5_ nanospheres.

The effect of the concentration of NbCl_5_ ethanol solution on the morphology and structure of hollow Nb_2_O_5_ nanospheres was investigated. At the relatively low concentration of NbCl_5_ ethanol solution (0.25 g/mL), insufficient niobium chloride enters the spherical pores of the carbon template, resulting in too little conversion of niobium oxide, and many niobium oxide fragments are generated (Figure 6a). Upon increasing the concentration of NbCl_5_ ethanol solution to 0.5 g/mL, the obtained niobium oxide is very similar to the carbon template, and spherical pores are interconnected with each other through small pores, but still, no niobium oxide nanospheres are produced (Figure 6b). When the concentration of the NbCl_5_ ethanol solution is further increased to 0.75 g/mL and 1.0 g/mL, hollow niobium oxide nanospheres are obtained (Figure 6c,d). The nanospheres are densely packed into larger particles, not monodisperse. The hollow nanospheres inside the particles are uniform in size, with smooth surfaces and perfect spherical shapes, while some of the hollow nanospheres on the outer surface of the particles have a broken structure, which is caused by the incomplete spherical holes on the outer surface of the carbon template particles. Taking into account that the higher the concentration of niobium chloride, the larger shell thickness and smaller specific surface area of the prepared niobium oxide nanospheres, the appropriate concentration of NbCl_5_ ethanol solution is 0.75 g/mL.

**Figure 7:**
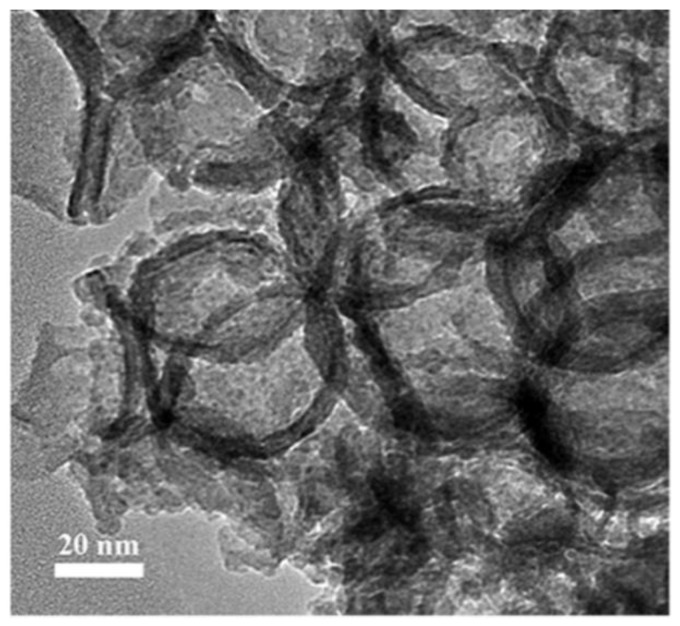
TEM image of hollow Nb_2_O_5_ nanospheres.

The TEM image of hollow Nb_2_O_5_ nanospheres in Figure 7 can further demonstrate that the prepared product has a hollow spherical structure with an outer diameter of about 45 nm and a shell thickness of 6 nm, which is consistent with the spherical pores of the carbon template. Because of the domain-limiting effect of the hard template, the prepared hollow Nb_2_O_5_ nanospheres have a much smaller diameter and shell thickness compared with those obtained using other preparation methods [11,19].

**Figure 8:**
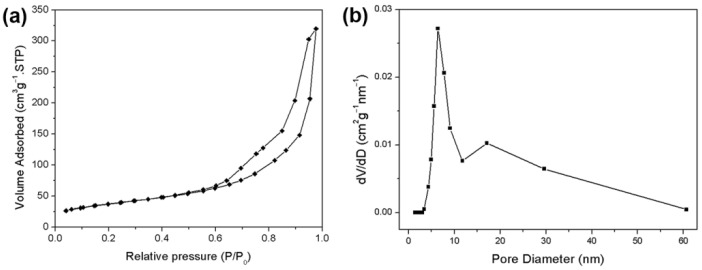
(**a**) N_2_ adsorption/desorption isotherms and (**b**) corresponding pore size distribution curve of hollow Nb_2_O_5_ nanospheres.

The nitrogen adsorption and desorption isotherms and the corresponding pore size distribution curve of the hollow niobium oxide nanospheres are presented in Figure 8. The nitrogen adsorption and desorption isotherms exhibit type IV curves with a clear hysteresis loop in a range of P/P_0_ greater than 0.5, indicating the mesoporous structures in the sample. There are two major regions where capillary condensation occurs at relative pressures of 0.5–0.85 and 0.85–0.95, which is a characteristic of the hierarchical porous structure. According to the pore size distribution curve, the pore size is mainly concentrated at 6 nm and also widely distributed in the range of 12 to 60 nm. The former corresponds to the through-hole contact between the sphere walls, while the latter corresponds to the stacked slit holes between the niobium oxide nanospheres. In addition, the specific surface area calculated by the BET method is 134.3 m^2^·g^−1^.

**Figure 9:**
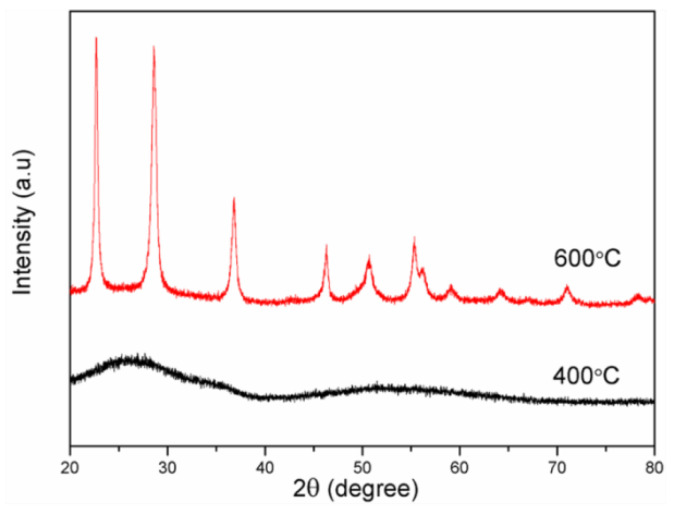
XRD patterns of hollow Nb_2_O_5_ nanospheres calcinated at different temperatures.

Figure 9 illustrates the XRD patterns of hollow niobium oxide nanospheres calcinated at different temperatures. The sample calcinated at 400 °C has only two diffraction peaks with weak and broad intensity and no crystalline phase indicated by sharp peaks, indicating that the sample is amorphous niobium oxide. When the calcination temperature was increased to 600 °C, the transformation of amorphous niobium oxide into the hexagonal crystalline phase occurred, which is consistent with JCPDS no. 07-0061 [20]. 

**Figure 10:**
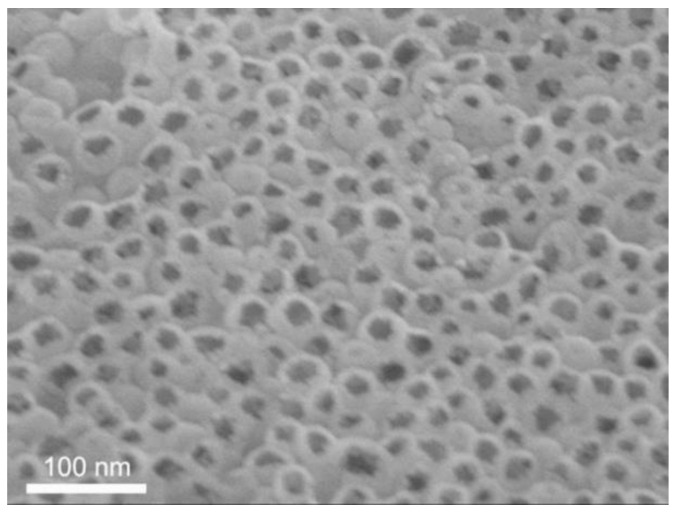
SEM image of hollow Nb_2_O_5_ nanospheres calcinated at 600 °C.

In addition, the morphology of the hollow niobium oxide nanospheres remained intact and undamaged in the case of higher temperature treatment (Figure 10), indicating that the prepared hollow niobium oxide nanospheres have good thermal stability. Hollow niobium oxide nanospheres with good crystallinity can be prepared through our preparation method. Moreover, elemental analysis (see Figure S2 in Supplementary Materials) by EDX shows that the prepared hollow mesoporous niobium oxide is composed of only two elements, niobium and oxygen (the C signal comes from the conducting resin), indicating that the prepared product is niobium pentoxide, and niobium chloride is completely converted into niobium oxide, while the silicon dioxide and carbon templates are completely removed.

### 3.2. Oxidative Desulfurization Performance of the Catalyst

**Figure 11:**
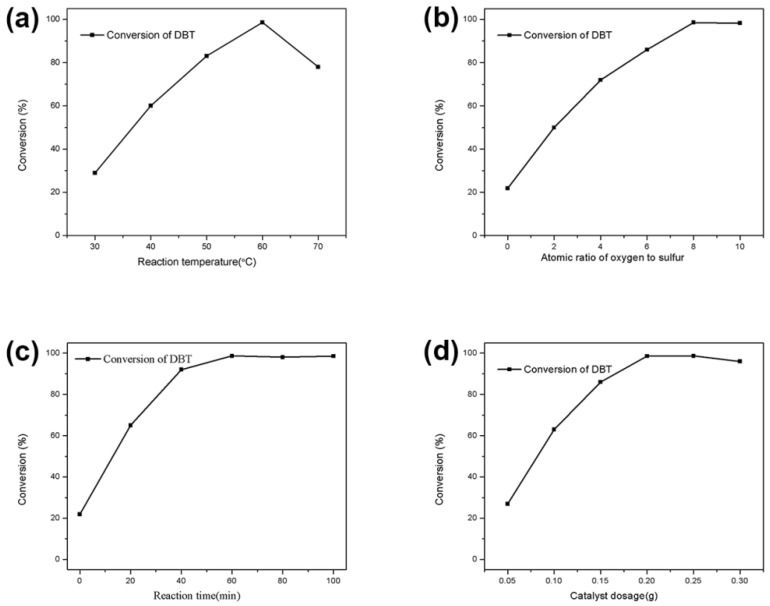
Effect of (**a**) reaction temperature, (**b**) oxygen/sulfur atomic ratio, (**c**) reaction time and (**d**) catalyst dosage on oxidation desulfurization of simulated oil. Reaction conditions: (**a**) oxygen/sulfur atomic ratio, 6; catalyst dosage, 0.2 g; reaction time, 1 h; simulated oil, 500 g; extractant, 200 g; (**b**) reaction temperature, 60 °C; catalyst dosage, 0.2 g; reaction time, 1 h; simulated oil, 500 g; extractant, 200 g; (**c**) reaction temperature, 60 °C; oxygen/sulfur atomic ratio, 6; simulated oil, 500 g; extractant, 200 g; (**d**) reaction temperature, 60 °C; oxygen/sulfur atomic ratio, 6; reaction time, 1 h; simulated oil, 500 g; extractant, 200 g.

In order to obtain suitable reaction conditions, the effects of reaction temperature, reaction time, oxygen/sulfur atomic ratio and catalyst amount on the oxidation desulfurization of simulated oil were systematically investigated (see Figure 11).

The catalytic oxidation desulfurization performance of commercial niobium oxide and hollow niobium oxide nanosphere catalysts on simulated oil was investigated under suitable conditions, with a reaction temperature of 60 °C, oxygen/sulfur atomic ratio of 6, catalyst dosage of 0.2 g, reaction time of 1 h and 500 g of simulated oil. The results are shown in Table 1. It can be seen from Table 1 that the catalytic activity of the hollow niobium oxide nanosphere catalyst is significantly higher than that of commercial niobium oxide for the catalytic oxidation desulfurization of simulated oil. The DBT conversion rate of the simulated oil is as high as 98.5% over the hollow niobium oxide nanosphere catalyst, which is better than that of commercial niobium oxide (49.6%). Due to the hollow structure and the hierarchical porous structure, the specific surface area of hollow niobium oxide nanospheres is as high as 134.3 m^2^·g^−1^, while the specific surface area of commercial niobium oxide is only 47 m^2^·g^−1^. The hollow niobium oxide nanosphere catalyst has a larger specific surface area, which usually provides more active sites for catalytic reactions. Additionally, the hierarchical porous structure can facilitate the diffusion of reactant molecules, making them more accessible to the active site [21]. Table 2 shows the oxidative desulfurization of DBT over various metal oxide catalysts using H_2_O_2_ as the oxidant. Compared with previously reported results, the prepared hollow niobium oxide also exhibits excellent catalytic performance in the elimination of DBT with the assistance of hydrogen peroxide among various metal oxide catalysts.

The reactivity of BT and 4, 6-DMDBT over hollow niobium oxide nanospheres was evaluated under the same conditions. As can be seen from Table 3, the conversion of sulfur compounds decreased in the order of DBT > BT > 4, 6-DMDBT. The lower conversion of BT and 4, 6-DMDBT may be due to the different electron densities of the sulfur atom and the steric hindrance of sulfur compounds. The electron densities on the sulfur atom of DBT, BT and 4, 6-DMDBT are 5.758, 5.739 and 5.760, respectively [25]. The conversion of DBT and BT decreased with decreasing electron densities on the sulfur atom. Although the electron density on the sulfur atom of 4, 6-DMDBT is the largest among the three sulfur compounds, the steric hindrance may hinder the accessibility of 4, 6-DMDBT to the active site due to the presence of two methyl substituents.

**Figure 12:**
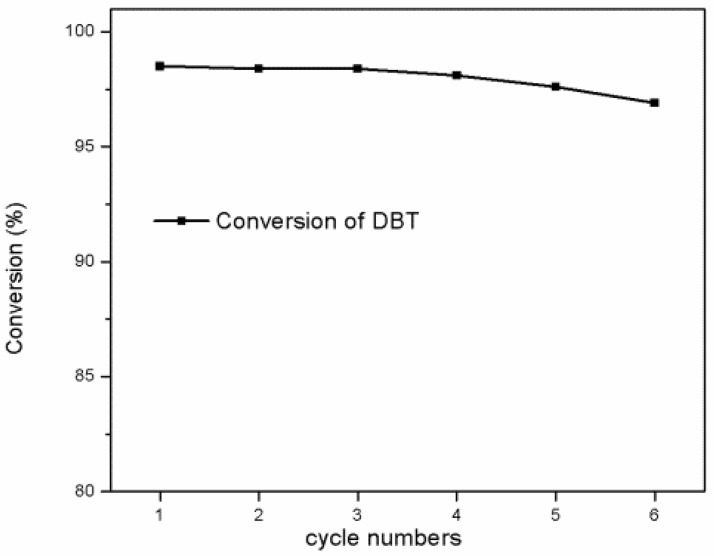
Reusability and stability testing of the hollow niobium oxide nanosphere catalyst.

For reusability and stability testing, the reaction was repeated for six cycles under similar conditions to those above. After each cycle, the catalyst was rinsed with ethanol, dried in the oven and used again for the next cycle. The results are summarized in Figure 12. The catalytic activity of the hollow niobium oxide nanosphere catalyst remains constant for the first three cycles and gradually decreases for the next three cycles. After repeated use for six cycles, the catalyst still has a high catalytic activity, and the conversion of DBT can reach 96.9%, indicating that the catalyst shows good reusability and high stability.

**Scheme 1:**
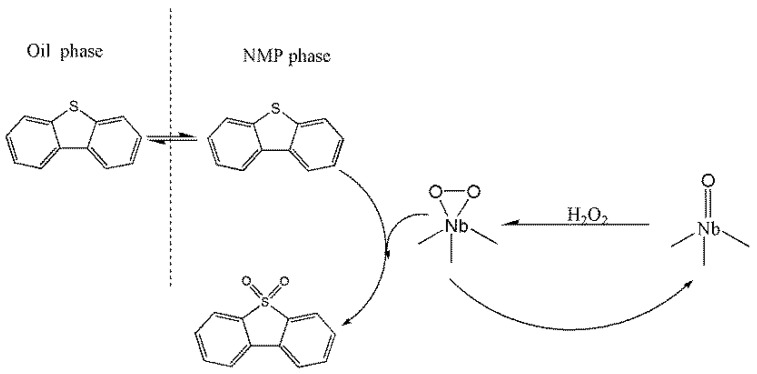
The possible reaction mechanism of oxidation desulfurization process.

The possible reaction mechanism is proposed in Scheme 1. DBT in the simulated oil is extracted to the solvent phase. Niobium peroxide species could be generated by nucleophilic attack on Nb=O by H_2_O_2_. Then, the sulfur atom in the organic sulfur compound nucleophilically attacks the active niobium peroxide species to form sulfones and regenerate niobium oxide.

## 4. Conclusions

(1) Three-dimensional mesoporous carbon materials were prepared by using histidine as the carbon source and silica microspheres as the hard template by adjusting the relative amounts of silica and histidine. Analysis using the nitrogen/adsorption measurement method showed that the three-dimensional mesoporous carbon materials have a typical porous structure with a high specific surface area of 893 m^2^·g^−1^.
(2) Hollow niobium oxide nanospheres were successfully synthesized by using 3D mesoporous carbon as the hard template and niobium pentachloride as the precursor with a vacuum-assisted method. The prepared product has a hollow spherical structure with an outer diameter of about 45 nm, which is consistent with the spherical pore size of the carbon template. The calculated specific surface area is as high as 134.3 m^2^·g^−1^.
(3) The oxidative desulfurization performance of the hollow niobium oxide nanosphere catalyst was investigated via the removal of DBT in simulated oil with hydrogen peroxide as the oxidant. Under suitable reaction conditions, the DBT conversion rate of the simulated oil was as high as 98.5%. The higher catalytic activity of the hollow niobium oxide nanosphere catalyst benefits from their larger specific surface area, unique hollow structure and hierarchical porous structure.
(4) The reusability and stability of the catalyst were evaluated by cycling tests, and a possible reaction mechanism is proposed.

## Figures and Tables

**Table 1:** The catalytic oxidation desulfurization performance of commercial niobium oxide and hollow niobium oxide nanosphere catalysts on simulated oil.

No.	Catalyst	DBT Conversion (%)
1	Commercial niobium oxide	50.3
2	Hollow niobium oxide	98.5

**Table 2:** Oxidative desulfurization of DBT over various metal oxide catalysts using H_2_O_2_ as oxidant.

Catalyst	T (°C)	Time (min)	DBT Conversion (%)	Ref.
MoO_x_@CN-1 (C/A = 1)	50	80	97.9	[22]
MoO_3_/γ-Al_2_O_3_	60	15	100	[9]
WO_3_ H_2_O	70	60	95.1	[23]
V_2_O_5_	30	150	98.7	[24]
Hollow Nb_2_O_5_ nanospheres	60	60	98.5	This work

**Table 3:** The reactivity of BT and 4, 6-DMDBT over hollow niobium oxide nanospheres.

No.	Sulfur Compounds	Conversion (%)
1	BT	84.3
2	4, 6-DMDBT	32.8

## Data Availability

All data, models and code generated or used during the study appear in the submitted article.

## Supplementary Materials

The following supporting information can be downloaded at: https://www.mdpi.com/article/10.3390/nano12071106/s1, Figure S1: SEM images of carbons prepared by different mass ratios of silica to histidine: (a,b) 1:0.5, (c,d) 1:1.0, (e,f) 1:1.5, (g,h) 1:2.0.; Figure S2: EDX pattern of the as-synthesized hollow Nb_2_O_5_ nanospheres.
