# Supplementary material for: Preparation of Hollow Niobium Oxide Nanospheres with Enhanced Catalytic Activity for Oxidative Desulfurization
Source: Nanomaterials (Basel). 2022 Mar 28;12(7):1106. doi: 10.3390/nano12071106 (PMC9000609; doi:10.3390/nano12071106)
Supplement: Supplementary file 1 [file nanomaterials-12-01106-s001.zip › nanomaterials-1642407-supplementary.pdf]

nanomaterials

Supplementary Materials

Preparation of Hollow Niobium Oxide Nanospheres with Enhanced Catalytic Activity for Oxidative Desulfurization

Yong Wang [1,*], Lei Ren [1], Zifeng Li [1] and Feng Xin [2]

[1] Sinopec Research Institute of Petroleum Processing, Beijing 100083, China; renlei.ripp@sinopec.com (L.R.); lizf.ripp@sinopec.com (Z.L.)
[2] School of Chemical Engineering and Technology, Tianjin University, Tianjin 300350, China; xinf@tju.edu.cn
* Correspondence: wangyong.ripp@sinopec.com; Tel.:+86-010-82368908

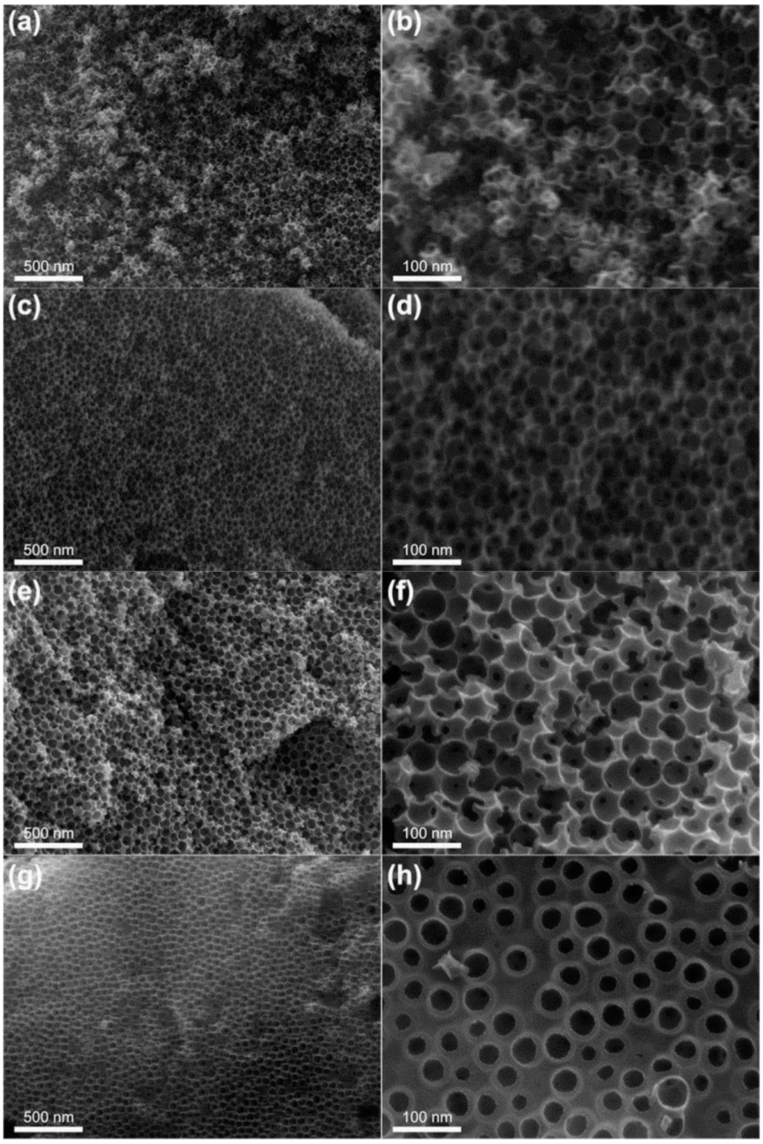

Figure S1. SEM images of carbons prepared by different mass ratios of silica to histidine (**a,b**) 1:0.5, (**c,d**) 1:1.0, (**e,f**) 1:1.5, (**g,h**) 1:2.0.

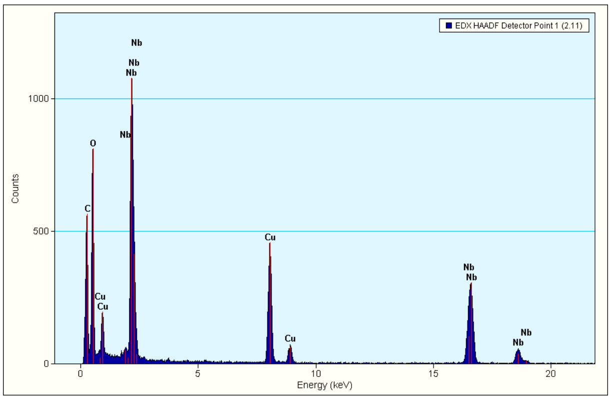

Figure S2. EDX pattern of the as-synthesized hollow Nb_2O_5 nanospheres.
